# A New Race of People in Russia

**Published:** 1882-07

**Authors:** 


### A NEW RACE OF PEOPLE IN RUSSIA.

In the *Revue Scientifique*, M. G. Le Bon treats at some length of a hitherto unknown people inhabiting an obscure part of Russia. Peculiar circumstances having induced the author to visit the Tatras Mountains, a very curious and beautiful region, and one very little known, since he was apparently the first to traverse it, he found there a territory surrounded on all sides by steep mountains and inhabited by a people speaking a different language from the nations surrounding them and with whom they had no intercourse. These people, although less than a century ago given up to brigandage, as the author learned in his study of them, are now very industrious and honest. In spite of a climate so harsh that it would be necessary to go to the extreme north to find a similar one, in spite of a very infertile soil, and in spite of an almost Lacedæmonian diet, consisting mainly of oats, milk, and water, they are living in a most remarkable state of prosperity. They are clearly distinguished from all their neighbors in their external aspect, in their quick intelligence, and in their artistic and literary tendencies.

The villages inhabited by these new people are situated in the territory called Podhale, at the foot of the above-named mountains. This territory, as before stated, being surrounded on all sides by steep mountains, difficult of access, is almost as isolated from the rest of the world as if it were an island in mid-ocean.

As regards its origin, Mr. Le Bon thinks the original stock was Polish, which in past ages became intermixed with individuals coming from different peoples. In isolating itself more and more, and not uniting with outsiders, and in constantly being submitted to the action of the same environment and of the same selection, the primitive agglomeration has become more and more homogeneous, and finally formed a new race, whose homogeneity may possibly still increase, but which already possesses common hereditary characters that permit it to be clearly differentiated from all surrounding races.

